# The effect of short-term intensive insulin therapy in newly-diagnosed Type-2 diabetic patients

**DOI:** 10.12669/pjms.37.7.4013

**Published:** 2021

**Authors:** Cengiz Karacaer, Taner Demirci, Hasret Cengiz, Ceyhun Varim, Ali Tamer

**Affiliations:** 1 Dr. Cengiz Karacaer Department of Internal Medicine, Sakarya University Treatment and Research Hospital, Department of Internal Medicine, Sakarya, Turkey; 2 Taner Demirci Assistant Professor, Department of Endocrinology and Metabolism, Sakarya University Treatment and Research Hospital, Department of Endocrinology and Metabolism, Sakarya, Turkey; 3 Dr. Hasret Cengiz Department of Endocrinology and Metabolism, Sakarya University Treatment and Research Hospital, Department of Endocrinology and Metabolism, Sakarya, Turkey; 4 Dr. Ceyhun Varim Department of Internal Medicine, Sakarya University Treatment and Research Hospital, Department of Internal Medicine, Sakarya, Turkey; 5 Ali Tamer Professor, Department of Internal Medicine, Sakarya University Treatment and Research Hospital, Department of Internal Medicine, Sakarya, Turkey

**Keywords:** Intensive insulin, Diabetes mellitus, Glycemic control, Beta-cell recovery, HbA1c

## Abstract

**Objectives:**

We aimed to determine the effect of short-term intensive insulin therapy (SIIT) on long-term glycemic control in newly-diagnosed Type-2 diabetes mellitus (nT2DM) patients.

**Methods:**

In this retrospectively study conducted at Sakarya University Medical Faculty Training and Research Hospital Outpatient Clinic between 2016 and 2019, 65 nT2DM patients were enrolled soon after their SIIT was initiated and were followed for at least a year. Intensive insulin treatment was discontinued after three or 12 months in a total of 65 (23–73-year-old) patients who had been newly diagnosed with T2DM. Intensive insulin therapy was discontinued when glycemic control and the target Glycated Hemoglobin (HbA1c) level had been attained, after which oral anti-diabetic drug (OAD), long-term insulin, and diet therapies were pursued.

**Results:**

There was a significant decrease in mean HbA1c from 11.25±1.96% to 6.67±1.07%. Fasting plasma glucose (FPG) was found to be an independent predictor of whether intensive insulin therapy could be discontinued after three months in a model that included FPG, HbA1c, and body mass index measured at baseline. Patients with FPG>13.8 mmol/L were 7.6 times more likely to require intensive insulin therapy beyond three months. There were significant decreases in HbA1c and low-density lipoprotein-cholesterol concentration, but no change in C-peptide between baseline and 3 months of therapy.

**Conclusion:**

These results demonstrate that in nT2DM patients, intensive insulin therapy could be effective on long-term glycemic control and high FPG prior to three months of SIIT may predict poor long-term glycemic control.

## INTRODUCTION

Type-2 diabetes mellitus (T2DM) is caused by an insulin deficiency that develops following chronic progressive beta-cell dysfunction. To prevent the deleterious effects of glucotoxicity and lipotoxicity on pancreatic beta cells, it is necessary to act to preserve the endogenous functionality of the pancreas as early as possible.^1^-^3^

An increase in circulating C-peptide concentration during the early stage of insulin therapy is the most important indicator of improvement in the endogenous beta-cell reserve.^3^ However, there have been few studies in which control was shown to be achieved using short-term intensive insulin therapy (SIIT) from as early as the first three months following diagnosis.^4^-^6^

A systematic review and meta-analysis recently demonstrated that 2–8 weeks of early, intensive insulin therapy can induce glycemic control, and after its discontinuation, patients can subsequently maintain glycemic control without anti-diabetic medication for up to 1–2 years.^7^

Reliable determinants of long-term glycemic control have yet to be determined. Therefore, we aimed to investigate the effects of SIIT on long-term glycemic control in newly diagnosed T2DM patients in this retrospective study.

## METHODS

The total number of patients followed up in the polyclinic was 4800, the number of patients with nT2DM was 122, and the number enrolled in the study was 65. In a study involving 65 patients with diet, exercise, OAD, and various insulin treatment modalities, patients treated with short-term intensive insulin therapy (SIIT) were included in the study. Patients diagnosed with T2DM according to the World Health Organization diagnostic criteria (1999) were selected for the study. Patients who applied to Sakarya University Medical Faculty Training and Research Hospital Outpatient Clinic between 2016 and 2019 and who had not received any diabetes treatment before were selected from the file records.

### Inclusion criteria:

1. 23–73 years old
2. Non-pregnant Type-2 diabetes mellitus
3. Have not had any previous diabetes treatment
4. Glycated Hemoglobin (HgbA1c) >8
5. Patients who were diagnosed with T2DM in the Diabetes Outpatient Clinic and came to the controls at three and 12 months

### Exclusion criteria:

1. Previous diabetes therapy,
2. Chronic renal or hepatic failure
3. Current malignancy
4. Chronic infection
5. Acute or severe chronic diabetic complications or other severe comorbidity
6. Pregnancy
7. Type-1 diabetes mellitus.

The study protocol was approved by the Ethics Committee of Sakarya University Medical Faculty and was conducted in accordance with the principles of the Declaration of Helsinki (71522473/050.01.04/166).

In this retrospective study patient data were collected from Diabetes Outpatient Clinic records. Demographic data, family history, chronic diseases, and medications were recorded from patient files retrospectively. Routine biochemical measurements made at baseline, including serum lipids and HbA1c, were recorded. Basic anthropometric measurements made using the Tanita TBF 300 device were recorded from the files. Laboratory tests previously performed by the patients in the Central Clinical Laboratory of Sakarya University Medical Faculty, Training and Research Hospital diabetes outpatient’s clinic were recorded from their files. In our diabetes outpatient clinic, all patients are given training programs on self-management of diabetes, including sports, lifestyle changes and food intake guidance.

### Intensive insulin therapy

Patients considered to be undergoing SIIT to reach their glycemic target (FPG 4.4–6.0 mmol/L, 2-hour post-prandial plasma glucose [PPG]: 4.4–7.8 mmol/L) were included in the study. Insulin Lispro (Humalog Kwikpen, Eli Lilly and Company, USA) or Insulin Aspart (Novorapid Flexpen, NovoNordisk, Bagsværd, Denmark) were the fast-acting analog insulins used. Insulin Glargine (Lantus Solostar, Sanofi Aventis, France) and Insulin Detemir (Levemir Flexpen Novo Nordisk, Bagsvaerd, Denmark) were the basal long-acting insulins used. Patients who administered a total daily insulin dose of 0.4–0.5 IU/kg, 50% of which was long-acting basal insulin and 50% fast-acting analog insulin were included in the study. The participants were taught to inject their short-acting insulin before each meal and their intermediate-acting insulin at bedtime. The type of insulin used was not included in the analysis.

### Blood sampling and measurement

In order to determine the ideal insulin dose required, a total of six blood glucose measurements were taken in the first few days, three before and three after meals (2 hours after the meal) in the basal bolus regimen of capillary glucose concentration. FPG, PPG, and HbA1c were measured monthly for three months after the cessation of SIIT. FPG 4.4-7.2 mmol and PPG 10 or <10 mmol/l were the targets for glycemic control, which are consistent with the American Diabetes Association (ADA) 2019 guidelines. All laboratory tests were performed in the Central Clinical Laboratory of Sakarya University Medical Faculty, Training and Research Hospital.

### Statistical Analysis

IBM SPSS Statistics version 23.0 (Armonk, NY, USA) was used for statistical analyses. Power analysis was applied. The Kolmogorov-Smirnov test was used to determine whether data distributions were normal. Non-normally distributed continuous data are presented using medians and interquartile ranges [IQRs]. Qualitative data are expressed as frequencies and percentages. The Friedman test was used to compare biochemical data among the three time points, and the Wilcoxon test was used for binary comparisons. The relationships between continuous variables were evaluated using multiple linear regression and Spearman correlation. Relationships between categorical variables were evaluated using multiple logistic regression (LR). Receiver operating characteristic (ROC) curve analysis was performed to determine the optimal cut-off for FPG. *P*< 0.05, was regarded as representing statistical significance.

## RESULTS

Sixty-five patients (41 men and 24 women), were recruited. Their mean age was 48.03 ± 10.78 (range 23–73 years). The mean FPG was 15.3± 6.1 mmol/L mean HbA1c 11.3 ± 1.96%, mean BMI 30.4 ± 5.8 kg/m^2^, respectively. All other laboratory and anthropometric data are presented in Tables-I & II.

**Table I:** Baseline clinical and TANITA parameters.

*Parameter*	*Baseline n(65)*
Gender (F/M)	24 (36.90)/41 (63.07)
Age (year)	48.03±10.78
Duration of DM (month)	1.21±0.53
Height (cm)	165.35±10.09
Weight (kg)	83.32±17.42
BMI (kg/m^2^)	30.48±5.81
TBMR (%)	1630.43±245.72
T1FAT (%)	30.83±10.87
FATMASS (kg)	30.83±10.87
TBW (kg)	40.42±7.31
FFM (kg)	55.21±9.98

DM: Diabetes Mellitus, BMI: Body Mass Index, TBMR: Total Basal Metabolisma Rate, T1 FAT: Total Body Fat Rate %, FAT MASS: Total Body Fat Mass), TBW: Total Body Water, FFM: Fat-Free Body Mass.

**Table II:** Comparisons of clinical characteristics between baseline, 2nd and 3rd visit.

*Parameters*	*Baseline [IQR:interquartile range]*	*2nd visit (3rd month) [IQR:interquartile range]*	*3rd visit (12 month) [IQR:interquartile range]*	*p*
N	65	57	65	
FPG (mmol/L)	15.3 [10.7 – 18.8]	6.1 [5.2 – 7.7]	6.3 [5.5 – 7.6]	<0.001 ^a,b^
PPG (mmol/L)	19.7 [14.8 – 23.3]	7.7 [6.9 – 9.7]	7.6 [6.6- 10.7]	<0.001 ^a,b^
HbA1c (%)	11.11 [9.8 - 12.3]	6.5 [5.9 - 7.3]	6.31 [5.8 - 7.2]	<0.001 ^a,b^
C-Peptide (pmol/ml)	2.28[1.41 - 5.46]	2.27 [1.42 - 6.54]	2.66 [1.42- 6.24]	0.240
LDL (mg/dl)	7.1 [5.7 - 9]	6.1[4.7 – 7.1]	6.2 [5.1 – 7.5]	0.001 ^a,b^
Creatinine (mg/dl)	0.86 [0.79 – 0.93]	0.78 [0.71 - 0.85]	0.78[0.72 - 0.83]	0.022 ^b^
ALT (U/L)	32.5 [24 - 53]	22.5 [16 - 29]	23.5 [18 - 37]	<0.001 ^a,b^
SAI (IU, dose/day)	24 [24 - 30]	26 [18 - 39]	31 [24 - 42]	0.416
SAI (IU, dose/kg)	0.33 [0.26 - 0.39]	0.35 [0.23 - 0.43]	0.4 [0.29 - 0.47]	0.841
LAI (IU, dose/day)	10 [10 - 14]	12 [10 - 16]	14 [10 - 16]	0.260
LAI (IU, dose/kg)	0.13 [0.11 - 0.18]	0.15 [0.11 - 0.2]	0.16 [0.13 - 0.21]	0.382
TID (IU, dose/day)	36 [32 - 40]	35 [24 - 49]	35 [18 - 50]	0.003 ^b^
TID (IU, dose/kg)	0.45 [0.38 - 0.56]	0.4 [0.28 - 0.62]	0.44 [0.23 - 0.61]	0.015 ^b^
IITD (month)	-	3 [1.5 - 3]	4 [2 - 6.5]	<0.001 ^a,b^
LAITD (month)	-	3 [2 - 3]	5 [2 - 7.5]	<0.001 ^a,b^

FPG: Fast Plasma glucose, PPG: post-prandial glucose; HbA1c: glycated hemoglobin A1c; LDL: Low density lipoprotein; ALT: Alanine aminotransferase; SAI: Short acting insulin; LAI: Long acting insulin; TID: total insulin dose; IITD: Intensive insulin treatment duration, LAITD: Long acting insulin treatment duration. Multiple comparison test (Wilcoxon test with Bonferroni adjustment) results: There was statistically significant difference between; a: baseline and 3rd month, b: baseline and 3rd visit.

Basically, the fasting plasma glucose (FPG) was 15.3 [10.8 – 18.8] and PPG was 19.7 [14.8 – 23.3]. Treatment with SIIT significantly reduced FPG and PPG at 3^rd^ and 12^th^ month (6.1 [5.2 – 7.7] at 3 months, 6.3 [5.4 – 7.6] at 12 months; *p*= 0.001) for FPG respectively and 7.7 [6.9 – 9.7] at 3 months, 7.6 [6.6 – 10.7] at 12 months *p*=0.001) for PPG respectively).

Basically, HbA1c was 11.11 [9.8 - 12.3], in the 3rd month 6.5 [5.9 - 7.3], in the 12th month 6.31 [5.8 - 7.2]; treatment with SIIT significantly reduced HbA1c at 3^rd^ and 12^th^ month (6.5 [5.9 – 7.3] at 3 months, 6.31 [5.8 – 7.2] at 12 months (*p*= 0.001) for HbA1c. LDL-cholesterol level (mg / dl) was significantly reduced to 6.1 [4.7 – 7.1] at 3 months and to 6.2 [5.1 – 7.5] at 12 months (*p*= 0.001) of SIIT therapy in 65 patients. The percentages of participants who had achieved their target HbA1c of < 7% were 67.3% after 3 months and 75% after 12 months.

Alanine aminotransferase (ALT) (U / L) basically 32.5 [24 - 53], 22.5 [16 - 29] at three months, 23.5 [18 - 37] at 12 months; IITD (month) 3 [1.5 - 3] at 3 months, 4 [2 - 6.5] at 12 months; LAITD (month) was found to be 3 [2 - 3] at 3 months, 5 [2 - 7.5] at 12 months (*p* = 0.001) (Table-II).

There was statistically significant positive correlation between delta total daily dose (ΔTDD) and fasting plasma glucose (FPG) (r: 0.418; p:0.003). There was statistically significant positive correlation between ΔTDD and short acting insulin (SAI) (r: 0.532; p:0.001). Additionaly, it was statistically significant positive correlation between long-acting insulin (LAI) (*r*= 0.608; *p=*0.001). There were statistically significant negative correlation between total daily dose (TDD-1) and fasting plasma glucose (FPG)(r:-0.376; p:0.011) and also it were positive correlation between SAI and LAI(r:0.986; p:0.001) (r:0.928; p:0.001) (Table-III).

**Table III:** Correlation of TDD-1 or ΔTDD with baseline clinical and laboratory parameters.

*Parameters*	*ρTHD (n=45)*		*THD-1(n=45)*	

	*r*	*p*	*r*	*P*
Age	0.169	0.250	0.311	0.375
Sex	-0.085	0.568	0.110	0.455
Weight (kg)	-0.114	0.441	0.218	0.137
BMI	-0.135	0.350	0.100	0.489
FPG	0.418	0.003	-0.376	0.011
HgbA1c (%)	-0.036	0.807	0.090	0.545
LDL-c	-0.150	0.310	-0.164	0.264
ALT	-0.208	0.166	-0.009	0.953
Crea	-0.247	0.094	-0.212	0.153
SAI	0.532	0.001	0.986	0.001
LAI	0.608	0.001	0.928	0.001
C-peptide	0.010	0.960	0.247	0.959

ΔTDD: Delta total Daily dose, BMI: Body Mass Index, FPG: Fasting Plasma Glucose, HbA1c: glycated hemoglobin, LDL-c: Low density lipoprotein cholesterol; ALT: Alanine aminotransferase; Crea: Creatinine, SAI: Short acting insulin; TID: Total insulin dose; IITD: Intensive insulin treatment duration, LAITD: Long-acting insulin treatment duration.

In the logistic regression analysis, it was found that the cessation of SIIT treatment after 3 months, FPG was an independent predictive factor (*p*=0.015) at the first admission, but HbA1c and BMI were not significant predictors.

**Fig.1:**
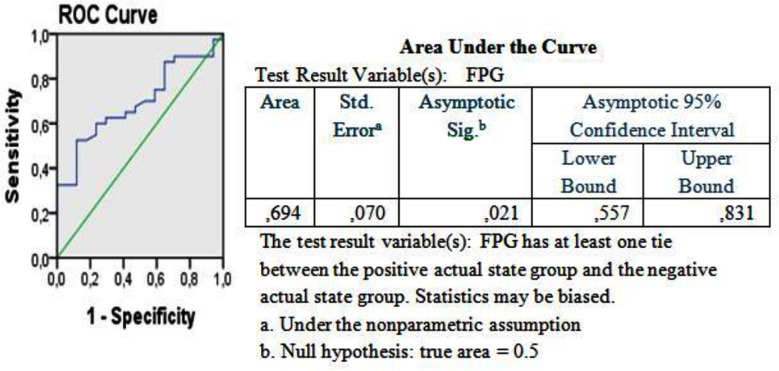
Examination of FPG value by ROC analysis in predicting t he cessation of insulin therapy and summary of analysis results. The ROC curve and reference diagonal line are shown together on the graph.

ROC analysis was performed to determine the optimal cut-off value of FPG for the determination of the requirement for insulin therapy beyond three months, and the area under the curve (AUC) was found to be significant (AUC: 0.694, 95% CI: 0.557–0.831, *p*=0.021). The use of FPG ≥ 13.8 mmol/L was associated with requirement for insulin therapy beyond three months and yielded a sensitivity of 62.5% and a specificity of 70.6% a LR analysis revealed that patients with FPG > 13.8 mmol/L were 7.6 times more likely to continue insulin therapy (*p*=0.011) (Fig.1, Table-IV).

**Table IV:** Whether the insulin therapy as the categorical variable in the logistic regression analysis should be discontinued or not and the probability of continuing or discontinuing insulin when FBG>250 mg/dL, BMI and HbA1c are used as the independent predictor factors.

	*B*	*Odds Ratio*	*95% C.I.*	*P*
FPG	0.011	1.011	1.002-1.020	0.015
BMI	0.069	1.072	0.949-1.211	0.266
HBA1c	-0.192	0.825	0.552-1.235	0.350

FPG: Fast Plasma Glucose, BMI: Body Mass Index.

**Fig.2:**
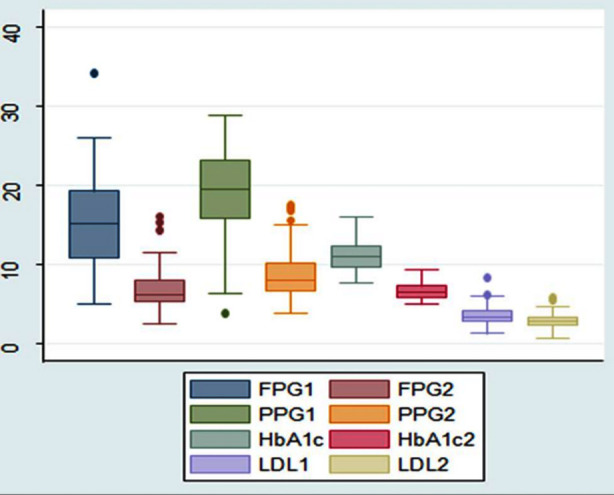
FPG1*-FPG2**, PPG1-PPG2, HbA1c1-HbA1c2, LDL1-LDL2 are proposed to be compared side-to-side(mmol/L). FPG: Fasting Plasma Glucose, PPG: Post Prandial Glucose, LDL: Low Dansity Lipid *1: Initial values of pre-treatment, **2: Third month values of treatment

Comparisons of data between two time points were performed using the Wilcoxon test (Fig.2). These showed that there were significant differences in FPG, PPG, and LDL-cholesterol level between baseline and 3 months (*p=*0.001) but that the C-peptide concentration did not differ (*p*=0.558). There was also a significant difference in creatinine concentration between baseline and 12 months (*p*=0.022).

The change in HbA1c during the first three months was close to significant following the application of the Bonferroni correction (*p*=0.05). There were significant differences in serum creatinine, total insulin dose, and insulin dose per kg between baseline and the second and third visits (Table-III).

## DISCUSSION

In the present study, we characterized the insulin requirements of 65 nT2DM patients who were treated using SIIT. The target HbA1c value after SIIT was achieved by 67.3% and 71.0% of participants after three months and 12 months, respectively. The initial insulin dose is 0.4-0.5 units/kg, making 33.5-41.5 units for an average weight of 83-90 kg. When euglycemia is reached, the daily insulin requirement is 36 [32-40] IU/day at baseline, 35 [24-49] IU/day after three months, 35 [18-50] IU/day after 12 months, and a constant basal/bolus ratio of 2: It was 3. Although the average insulin doses do not appear to have decreased, it should be noted that the minimum insulin doses gradually decreased at the second and third visits. (*p*=0.03) (Table-II).

To date, the proposed predictors of diabetic remission include the blood glucose concentration shortly after therapy, improvements in indices of beta-cell function, a reduction in insulin resistance, compliance with lifestyle modification, and a positive attitude towards the management of hyperglycemia.^8^,^9^

We have shown significant reductions in FPG, short acting insulin dose, and long acting insulin dose in correlation analyses performed *using* data from the 69% (45 of 65) of patients who had entered remission after 3 months of SIIT (*p* = 0.011, *p* = 0.001, and *p* = 0.001, respectively).In the study of Kramer CK et al. and Chen A et al., it were shown that short-term intensive insulin therapy (SIIT) correlated with improvement in FPG in long-term outcomes in nT2DM patients.^10^,^11^

In the previous studies, FPG has been reported to have a predictive role in predicting the onset of diabetes, even if values are within the normal range.^8,12-13^ Ozery-Flato M et al. in their study, it was shown that they can accurately predict the development of T2DM with measurements such as BMI, FPG and HbA1c.^14^

In our study, although FPG lower than 13.8 mmol/L is an independent predictive factor indicating that short term intensive insulin therapy can be cessation, BMI and HbA1c were not found to be an independent predictive factor. In previous studies, there are studies that reached this conclusion in a period of 6-12 months.^15^ There is a lack of consensus on the optimal duration of insulin therapy. Our study has provided information that the initial blood glucose can give an idea about the optimal duration of intensive insulin therapy in patients with newly diagnosed Type-2 diabetes who are metabolically glucotoxic.

Long-term insulin use has concerns regarding hypoglycemia, weight gain, decreased patient compliance and potential malignancy.^16^-^18^ This indicates that more effective predictive factors are needed to achieve glycemic control.

We found that FPG, PPG, HbA1c, LDL-cholesterol, and ALT were significantly lower after 3 and 12 months than at baseline. However, no change in the C-peptide concentration was detected (*p*=0.240). Insulin action can be impaired by chronic hyperglycemia, but this defect can be ameliorated by the establishment of euglycemia. In the present study, an FPG of 9.2 mmol/L, a PPG of 11.9 mmol/L, and an HbA1c of 5.6% were found to be predictors of a recurrence of hyperglycemia (Table-II). We have shown that rapid correction of hyperglycemia can greatly improve β-cell function, especially with respect to first-phase insulin secretion. Therefore, in patients with nT2DM, in whom there has been a shorter duration of glucotoxicity and lipotoxicity, the early impairments in β-cell function can be reversed, which permits the long-term maintenance of glycemic control. In one study, HbA1c was significantly associated with various lipid parameters, and the importance of glycemic control in managing dyslipidemia and further reducing cardiovascular diseases risk in patients with T2DM was emphasized.^19^

SIIT has been used to obtain more rapid and better glycemic control in nT2DM patients, but predictors of long-term glycemic control have yet to be identified. Therefore, in nT2DM patients requiring insulin, we conducted a retrospective study that aimed to determine whether SIIT performed during the first 3 months after diagnosis would improve long-term glycemic control and ameliorate risk factors.

### Limitations of the study

The major limitation of our study were lower sample size, retrospective design and the short-term follow-up period.

### Strength of the study

It includes the high rate of improvement in FPG and the fact that this predictive factor was determined in the ideal period of 3 months is what makes it superior to other studies. The present study was designed to identify predictors of success in achieving glycemic control. However, the results require confirmation in larger studies before they can be applied in a routine clinical setting. Comparing the efficacy of intensive dietary therapy and aggressive therapy with insulin or OADs will be required in further studies.

## CONCLUSION

The present study aimed to identify independent predictors of the need to prolong insulin therapy beyond three months. We found that FPG was an independent predictor in a model constructed using FPG, HbA1c, and BMI values very early in the course of nT2DM. β-cell function improved significantly during the first three months of SIIT, as did glycemic control. Our study shows that SIIT improves β-cell function and insulin sensitivity in patients with nT2DM.

### Author’s contribution:

***CK:*** Conceived the study, data collection, writing manuscript and responsible for integrity of study.

***HC:*** Helped to draft the manuscript.

***TD:*** Preparation of the manuscript.

***CV:*** Editing and final approval.

***AT:*** Study design.

All authors read and approved the final manuscript.
